# Transcriptomics of Desiccation Tolerance in the Streptophyte Green Alga *Klebsormidium* Reveal a Land Plant-Like Defense Reaction

**DOI:** 10.1371/journal.pone.0110630

**Published:** 2014-10-23

**Authors:** Andreas Holzinger, Franziska Kaplan, Kathrin Blaas, Bernd Zechmann, Karin Komsic-Buchmann, Burkhard Becker

**Affiliations:** 1 University of Innsbruck, Functional Plant Biology, Innsbruck, Austria; 2 Baylor University, Center for Microscopy and Imaging, Waco, Texas, United States of America; 3 University of Cologne, Botanical Institute, Biocenter, Cologne, Germany; Universidade Federal de Vicosa, Brazil

## Abstract

**Background:**

Water loss has significant effects on physiological performance and survival rates of algae. However, despite the prominent presence of aeroterrestrial algae in terrestrial habitats, hardly anything is known about the molecular events that allow aeroterrestrial algae to survive harsh environmental conditions. We analyzed the transcriptome and physiology of a strain of the alpine aeroterrestrial alga *Klebsormidium crenulatum* under control and strong desiccation-stress conditions.

**Principal Findings:**

For comparison we first established a reference transcriptome. The high-coverage reference transcriptome includes about 24,183 sequences (1.5 million reads, 636 million bases). The reference transcriptome encodes for all major pathways (energy, carbohydrates, lipids, amino acids, sugars), nearly all deduced pathways are complete or missing only a few transcripts. Upon strong desiccation, more than 7000 transcripts showed changes in their expression levels. Most of the highest up-regulated transcripts do not show similarity to known viridiplant proteins, suggesting the existence of some genus- or species-specific responses to desiccation. In addition, we observed the up-regulation of many transcripts involved in desiccation tolerance in plants (e.g. proteins similar to those that are abundant in late embryogenesis (LEA), or proteins involved in early response to desiccation ERD), and enzymes involved in the biosynthesis of the raffinose family of oligosaccharides (RFO) known to act as osmolytes). Major physiological shifts are the up-regulation of transcripts for photosynthesis, energy production, and reactive oxygen species (ROS) metabolism, which is supported by elevated cellular glutathione content as revealed by immunoelectron microscopy as well as an increase in total antiradical power. However, the effective quantum yield of Photosystem II and CO_2_ fixation decreased sharply under the applied desiccation stress. In contrast, transcripts for cell integrative functions such as cell division, DNA replication, cofactor biosynthesis, and amino acid biosynthesis were down-regulated.

**Significance:**

This is the first study investigating the desiccation transcriptome of a streptophyte green alga. Our results indicate that the cellular response is similar to embryophytes, suggesting that embryophytes inherited a basic cellular desiccation tolerance from their streptophyte predecessors.

## Introduction

Poikilohydric plants such as algae do not have protective structures (such as a waxy cuticula) and cannot actively regulate the transpiration rate (e.g. by stomata), which can easily lead to desiccation under water-limited conditions. Desiccation tolerance of these organisms can be defined as the ability to survive drying to ∼10% remaining water content, which is equivalent to ∼ 50% relative air humidity (RH) at 20°C [1], [2]. Aeroterrestrial green algae are naturally exposed to this stress, which they must tolerate in order to survive and propagate under these conditions (for a recent summary see [3]). The mechanisms to tolerate dehydration might involve the formation of permanent stages (e.g. akinetes, zygospores; e.g. [4]), but dehydration might also be tolerated in the vegetative state. Desiccation-tolerant species of green algae have been found in phylogenetically distinct lineages, e.g. in the so-called lichen algae, Trebouxiophyceae (e.g. [5], [6]), but also in streptophyte green algae, which represents the sister lineage to land plants (e.g. [7]–[11]).

A primary target of dehydration in green algae is photosynthesis (for a summary see [3]). Early studies reported an effect on CO_2_ exchange in the Trebouxiophyte *Apatococcus lobatus* [12]. While the most favourable carbon assimilation was measured in *A. lobatus* at ∼98% RH, even at RH 90%, 50% of the maximum CO_2_ uptake still occurred. The lower limit of carbon assimilation was observed at 68% RH [12]. More recently, Gray et al. [13] used ambient air at 25% RH to desiccate various strains of algae isolated in deserts and their aquatic relatives from the Chlorophyceae (e.g. *Bracteacoccus*, *Scenedesmus*, *Chlorogonium*) and Trebouxiophyceae (e.g. *Chlorella*, *Myrmecia*). They found that the desert algae could survive 4 weeks of desiccation when dried in darkness, and recovered photosynthetic quantum yield within 1 h of rehydration. Resurrection kinetics were also studied in other desiccation-tolerant Chlorophyta (e.g. *Desmococcus*, *Apatococcus*, *Trebouxia*), where green biofilm samples underwent prolonged desiccation (up to 80 days) and upon rehydration recovered about half of the initial value prior to desiccation [6]. In searching for the mechanisms behind this tolerance, Wieners et al. [14] observed desiccation-induced non-radiative dissipation in isolated green lichen algae (*Trebouxia*). Dynamic photoinhibition has been confirmed for several species of desert and aquatic algae (e.g. *Klebsormidium*, *Cylindrocystis*, *Stichococcus*; [15]). Photoprotective mechanisms were also found in the investigated algal strains; however, lineage-specific modifications occurred [15].

In *Klebsormidium crenulatum* [16]–[18] and a related *Klebsormidium* species [19]–[20], the optimum quantum yield decreased to zero after the cells were desiccated at ambient room temperature and RH; the exact percentage decrease was not determined, but recovered to different extents quickly after rehydration [16], [19]. Differences in desiccation tolerance might be also important for intra-generic ecological differentiation. For example, Škaloud and Rindi [21] suggested the existence of an ecological differentiation in *Klebsormidium,* according to different water-holding capacities of the substrates. However, on the global scale populations of *Klebsormidium* were described as genetically homogenous, and the local genotypes may be caused by ecological factors such as habitat differentiation [22]. Phylogenetic relationships within the genus *Klebsormidium* were recently elucidated (e.g. [20]–[21], [23], [24]), and the strain of *K. crenulatum* investigated here has been characterized by its *rbc*L gene [18] and is belonging to clade F according to [24].

Although some information is available on physiological adaptations to desiccation in green algae, no molecular approach has been used so far to understand desiccation tolerance in these organisms. Proteome analyses of the Trebouxiophycean green alga *Asterochloris erici*, a lichen-forming organism, found that only a few proteins increase upon desiccation [25]. It was not possible to identify changes in protein content involved in the light reactions of photosynthesis; however, dehydration led to a decrease in proteins associated with the Calvin cycle, indicating a reduction in carbon fixation. Interestingly, *A. erici* is still able to maintain initial values of maximum and actual quantum yield until 20% relative water content is reached [5]. Presumably, the loss of certain proteins does not halt carbon fixation during drying [25].

Deep sequencing (Illumina RNA-seq) provides a fast and reliable approach to generate large expression databases for functional genomic analysis, and is particularly suitable for non-model species with unsequenced genomes (e.g. [26]–[28]). While the analysis of the plant transcriptomes has produced an unexpected abundance of data on transcript identity in vascular plants, including resurrection plants (e.g. [29]–[31]), little information is yet available on transcriptomes in algae. In the unsequenced microalga *Chlorella vulgaris*, triacylglycerol biosynthetic pathways were analyzed by a transcriptome approach [32]. In *C. vulgaris* Illumina sequencing produced 27 million reads, Velvet and Oasis program suites were utilized for *de novo* transcriptome assembly, yielding 29,237 transcripts with an average length of 970 b. The results suggested an up-regulation of fatty-acid and triacylglycerol-biosynthetic mechanisms under nitrogen limitation [32]. A study using brown algal kelp *Saccarina latissima* identified transcriptional changes upon temperature and light stress; 32% of the genes showed an altered expression under the experimental conditions used [33]. Heinrich et al. [33] also found that high temperatures had a stronger effect on gene expression than low temperatures. The molecular processes of acclimation included the adjustment of the primary metabolism, induction of several ROS scavengers and regulation of heat-shock proteins (HSPs) [33]. A combination of high temperatures and high light intensities proved to be most harmful to this kelp species. Another study on the kelp *Saccharina japonica* investigated the effect of blue light on the transcriptome [34]. Their RNA-seq analysis yielded more than 70,000 non-redundant unigenes with an average length of 538 bp, and 40.2% of these could be mapped to transcript-encoding regions. Upon blue light exposure, ∼7,800 unigenes were up-regulated and ∼3,850 down-regulated. The feasibility of next-generation sequencing in the green algae *Coleochaete* and *Spirogyra* has been shown by Timme and Delwiche [35], and more than 30 transcriptomes were sequenced in the 1KP Project (http://onekp.com/project.html). The sequenced charophyte green algal transcriptomes have been shown to be remarkably similar to land plants, which demonstrate the evolutionary significance of these studies. Currently about 60,000 expressed sequence tags (ESTs) are available in GenBank for *Klebsormidium* (NCBI, 22.07.2014), representing several different species and strains. However, so far no studies on the effect of desiccation on the transcriptome of a streptophyte green alga are available.

In the present study, we exposed laboratory-grown *Klebsormidium crenulatum* to a strong desiccation stress in order to study, at the transcriptome level, the key molecular processes required for desiccation tolerance of this alga. To corroborate our findings, we performed physiological measurements of CO_2_ assimilation rates, effective quantum yield and antiradical power, and determined the cellular glutathione levels by immunoelectron microscopy. This enabled us to discuss the transcriptome findings in the context of the cellular strategies against desiccation.

## Material and Methods

### Algal cultures

*Klebsormidium crenulatum* (Kützing) Lokhorst [36] SAG 2415, previously isolated from an alpine soil crust [16], were cultivated in modified Bold's basal medium (3NMBBM (triple nitrate concentration [37]) or Waris-medium [38]) at 30–25 µmol photons m^−2^s^−1^ in a light:dark regime of 16∶8 in an Intellus environmental controller (Percival Scientific, Perry, IA, USA) at 20°C as previously described [16], [18]. The phylogenetic position of this strain was determined by *rbc*L sequences, deposited in GenBank under accession number JN190354 [18].

### Desiccation experiment

Two-week-old cultures of *K. crenulatum* were concentrated by vacuum filtration onto S&S mixed cellulose membrane filters (Sigma Aldrich Z 612383, pore size 0.45 µm) in triplicate (n = 3). The experiment was carried out 2 h after onset of light. The filters were then desiccated on top of 4 glass columns holding a perforated metal grid inside a 200 mL polystyrol box over ∼ 100 g silica gel (Silica Gel Orange, Carl Roth, Karlsruhe, Germany) for 2.5 h according to the methods of [39]. During this procedure, the temperature and the relative air humidity in the box were monitored with a PCE-MSR145S-TH mini data logger for air humidity and temperature (PCE Instruments, Meschede, Germany). The weight of the mixed cellulose membrane filters was determined prior to vacuum filtration. Then the weight of the fresh biomass (after filtration of the algae; cells and surrounding water film) and the weight of the desiccated biomass (after 2.5 h of desiccation over silica gel) were determined. The filter weight was subtracted to gain the net weight of the fresh or desiccated biomass, respectively. The reduction (%) of the water content of the fresh biomass was calculated following the equation: 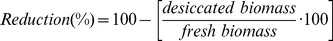



The desiccated biomass was then dried at 100°C for 13 h. The relative water content (RWC; %) of the desiccated biomass was calculated following the equation:
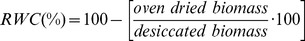



### Staining with FM 1–43

Control cells, cells desiccated for 2.5 h, and cells allowed to recover for 2 h in culture medium after desiccation were stained with a 40 µM solution of FM1–43 (green biofilm cell stain, Invitrogen Ltd., Paisley, UK, prepared from a 20 mM stock solution in DMSO). Cells were observed directly in the staining solution in a Zeiss Pascal confocal laser scanning microscope (Carl Zeiss AG, Jena, Germany). Samples were excited with an argon laser beam (488 nm), and the emission was collected in two separate channels at a wavelength band between 505–550 nm (false-colored green) and at wavelengths longer than 560 nm (false-colored red).

### Pulse amplitude fluorescence (PAM) and gas exchange measurements

The effective quantum yield (ΔF/Fm’) of photosystem II (Yield II) was determined every 10 min during the dehydration period, using a pulse-amplitude modulated fluorimeter (PAM 2500, Heinz Walz GmbH, Effeltrich, Germany) according to the methods described by [39]. ΔF/Fm’ was calculated as (Fm’-F)/Fm’ with F as the fluorescence yield of light-exposed algal cells (40 µmol photons m^−2^ s^−1^) and Fm’ as the maximum light-adapted fluorescence yield after employing an 800 ms saturation pulse, as described by Schreiber and Bilger [40]. The PAM light probe was positioned outside the desiccation box at a distance of 2 mm. The measurements were carried out on 3 individual filters (n = 3) containing algal suspension, placed in individual desiccation boxes. At each filter 3 measurement points were recorded every 10 min. The CO_2_ assimilation rate of *K. crenulatum* was measured by a GFS-3000 portable gas exchange fluorescence system (Heinz Walz GmbH, Effeltrich, Germany) with a LED-Array/PAM-Fluorometer 3055-FL under strong desiccation stress at ∼20% RH (n = 3) for 2.5 h. 100 mL homogeneous algal culture was filtered on a ME25 membrane filter (mixed cellulose ester, Whatman GmbH, Germany). Cells were removed from the filter areas that were not placed inside the measurement chamber. The assimilation rate was calculated based on the area (8 cm^2^) of the measurement chamber. After a recovery period in culture medium overnight (21 h), the assimilation rate was monitored again. Standard conditions for all measurements were 20°C, 30 µmol photons m^−2^ s^−1^, 380 ppm absolute CO_2_ content and a molar flow rate at the inlet of the cuvette of 750 µmol s^−1^. Absolute water content was calculated with a Vaisala Humidity Calculator 1.3 (Vaisala, Helsinki, Finland) to adjust the required relative humidity in the GFS-3000 measurement chamber to 4919 ppm for 20% RH. Assimilation rate was measured at 1-min intervals, and in parallel Yield II was measured at 3-min interavls. After each measurement, the chlorophyll a content of the sample was determined. Chlorophyll a was extracted in dimethylformamide (DMF) and quantified according to Porra at al. [41]. Values of the initial 5 min of the desiccation period, of the desiccated sample, and of the initial 5 min of the recovery measurements were statistically analyzed with SPSS 15.0 for Windows (IBM Corporation, Somer, NY, USA), using one-way ANOVA followed by Tukeýs post-hoc test (p≤0.001).

### RNA isolation

RNA was isolated from *K. crenulatum* cells either directly concentrated on mixed cellulose filters or desiccated as described above. The filters were swiped with 450 µL of Life Guard Soil Preservation solution (MO BIO Laboratories, Inc., Carlsbad, CA, USA, Cat No. 12868-100). The cells were frozen in liquid nitrogen, then ground with a mortar and pestle, followed by RNA extraction using a PEQLAB Gold Plant RNA isolation kit (PEQLAB, Erlangen, Germany) according to the manufacturer's instructions. For DNA removal the RNA was treated with DNaseI (Thermo Scientific, DNaseI, RNase free). A final cleanup was performed, again using the PEQLAB RNA column.

### Expression analysis

Random primed cDNA libraries were prepared by GATC Biotech AG (Konstanz, Germany). The library preparations were sequenced on an Illumina HiSeq 2000 as single-reads to 100 bp in the same flow cell (first sequencing run). All sequences were analyzed using the CASAVA v1.7 (Illumina, USA). To establish a reference transcriptome, the same amounts of RNA from the control and desiccated samples were pooled and a normalized random primed cDNA library was prepared from the pooled samples by GATC Biotech AG (Konstanz, Germany). The reference library was sequenced on a Roche GS FLX (1/2 plate), yielding 1,553,953 sequenced reads (636,002,000 sequenced bases). Assembly and basic expression analysis were performed at GATC Biotech AG. Briefly, the reference genome library was assembled using the De Novo Assembler (Newbler) 2.8 software from Roche. For expression analysis, sequence reads were mapped to the reference sequence (unigene.highcov.fa) using BWA with the default parameters. Only uniquely mapped reads were considered for further processing. PCR duplicates were removed using PICARD. The resulting high-quality sequence alignments were taken for coverage determination and variant detection. The alignments were processed to compute the read counts for each exon/CDS/transcript. Read counts were then normalized to 1 million reads (RPM) by considering the total reads mapped in each sample, to remove the uneven sequencing coverage over multiplexed samples. Finally, RPM counts were normalized to 1 Kbp transcript length (RPKM) to remove the uneven gene/transcript length bias. The final table included all the transcripts present in the reference employed. The entries were filtered based on defined threshold before doing the expression analysis. Determining counts, RPM and RPKM computations were done using in-house scripts by GATC-Biotech.

For differential expression analysis, the DeSeq software [42] was used, following the program's vignette (DESeq version 1.12.0, Last revision 2013-02-24). Pathways and modules were analyzed using the KEGG module and pathway databases at http://www.genome.jp/kegg/. Local Blast analyses were performed using the NCBI BLASTX program and the protein databases from the following species obtained at www.phytozome.net: *Chlamydomonas reinhardtii, Coccomyxa subellipsoidea, Micromonas pusilla, Ostreococcus lucimarinus, Physcomitrella patens, Selaginella italica, Zea mays, Oryza sativa, Aquilegia coerulea, Solanum tuberosum, Eucalyptus grandis, Theobroma cacao, Arabidopsis thaliana, Malus domesticus, Medicago trunculata* and *Populus trichocarpa* RNA-Seq data and the assembled contigs have been deposited in the NCBI's Gene Expression Omnibus (GEO) [43] and are accessible through the NCBI Sequence Read Archive (http://trace.ncbi.nlm.nih.gov/Traces/sra/) under the accession numbers SRR1514242 (assembled reference library) and SRR1563130 to SRR1563134 (transcriptomes).

### Cytohistochemical detection of glutathione

Preparation of samples for transmission electron microscopy and immunogold labeling of glutathione was performed as previously described [44], [45]. Briefly, algae were fixed in 2.5% paraformaldehyde and 0.5% glutardialdehyde in 0.06 M phosphate buffer (pH 7.2), rinsed in the buffer, dehydrated in increasing concentrations of acetone (50%, 70%, and 90%) and infiltrated with increasing concentrations of LR-White resin (30%, 60% and 100%; London Resin Company Ltd., Berkshire, UK). Samples were polymerized at 50°C. Ultrathin sections were blocked with 2% bovine serum albumin (BSA) in phosphate buffered saline (PBS, pH 7.2) and treated with the primary antibody (anti-glutathione rabbit polyclonal IgG, Millipore Corp., Billerica, MA, USA) diluted 1∶50 in PBS containing 1% goat serum. After three brief rinses in PBS, sections were incubated with 10 nm gold-conjugated secondary antibodies (goat anti-rabbit IgG for glutathione, British BioCell International, Cardiff, UK) 1∶50 in PBS and finally washed with distilled water. A minimum of 25 different cells were analyzed for gold particle density. The data obtained were presented as the number of gold particles per μm^2^.

### DPPH-Test

For determination of the antiradical power of fresh and 2.5 h desiccated *K. crenulatum* cells, a 2,2-diphenyl-1-picrylhydrazyl (DPPH)-test using 6-hydroxy-2,5,7,8-tetramethylchroman-2-carbonacid (Trolox, Sigma Aldrich, Germany) as standard was used according to the methods of [46]. *K. crenulatum* cultures were filtered according to the above-mentioned procedure onto Whatman GF/F glass fiber filters (Whatman, Dassel, Germany) and wrapped in aluminum foil, either immediately as a control (n = 3) or after desiccation for 2.5 h over silica gel (n = 3). Filters were then frozen in liquid nitrogen, and stored at −80°C prior to lyophilization. Lyophilized algae were then extracted in plastic vials containing 5 mL of 70% acetone for 24 h at 4°C under continuous shaking in darkness. 200 µL of a 150 µM DPPH-solution in 80% ethanol was transferred into a 96-well microtiter plate. The DPPH solution was complemented with 22 µL of the respective samples or Trolox standards in 70% acetone (7.8 µM-1 mM). After 25 min the absorption was determined for each sample at 516 nm. The statistical significance of means was tested by two-sample *t*-test.

## Results

### Experimental setup and physiological response to desiccation stress in *Klebsormidium crenulatum*

**Figure 1:**
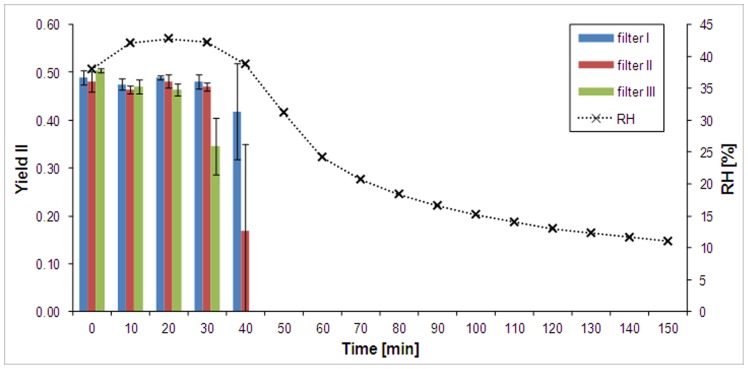
Effective quantum yield of photosystem II (Yield II) of *Klebsormidium crenulatum* during desiccation (desiccated over 100 g of silica gel) monitored by PAM 2500. Values represent means (n = 3), error bars show standard deviation (SD). The results of three filters containing similar amount of algal biomass are shown individually, to demonstrate that the decrease of Y II occurred within a short time frame (40–50 min). The relative air humidity (RH) inside the chamber (right y-axis) was monitored by a PCE sensor.

To identify the molecular processes involved in the desiccation response in *K. crenulatum,* filaments were transferred to filter membranes and incubated for 2.5 h in a desiccation chamber over silica gel. At the end of the experiment, the relative humidity in the desiccation chamber was ∼10% (Fig. 1). The weight of the fresh biomass (algae and surrounding water film) was reduced by 95.16±1.41% during the desiccation process. The experimentally desiccated algae had a RWC of 6.54±1.89%.

Within 40–50 min after exposure of the filters to silica gel, the effective quantum yield of *K. crenulatum* cells dropped to zero, suggesting complete inhibition of photosystem II (Fig. 1). When rehydrated with medium after the 2.5 h desiccation period, the effective quantum yield of photosystem II fluorescence did not recover for at least 2.5 h (not shown). Gas exchange measurements confirmed this observation (Fig. S1). The initial CO_2_ assimilation rate was ∼0.5 µmol CO_2_ m^−2^ s^−1^ at the beginning of the measurements (Fig. S1). CO_2_ assimilation of photosynthesis was inhibited immediately when the RH dropped (Fig. S1). At the end of the desiccation period, a net respiration was found. Cells did not recover completely when the filters were rewetted, and the measurements continued; after a 21-h recovery period, respiration still predominated, although at a lower level (−0.05 to −0.1 CO_2_ m^−2^ s^−1^). In agreement with the measurements from the desiccation chamber (Fig. 1), and also in the gas-exchange measurements, the effective quantum yield of photosystem II (Yield II, Fig. S1) did not recover.

**Figure 2:**
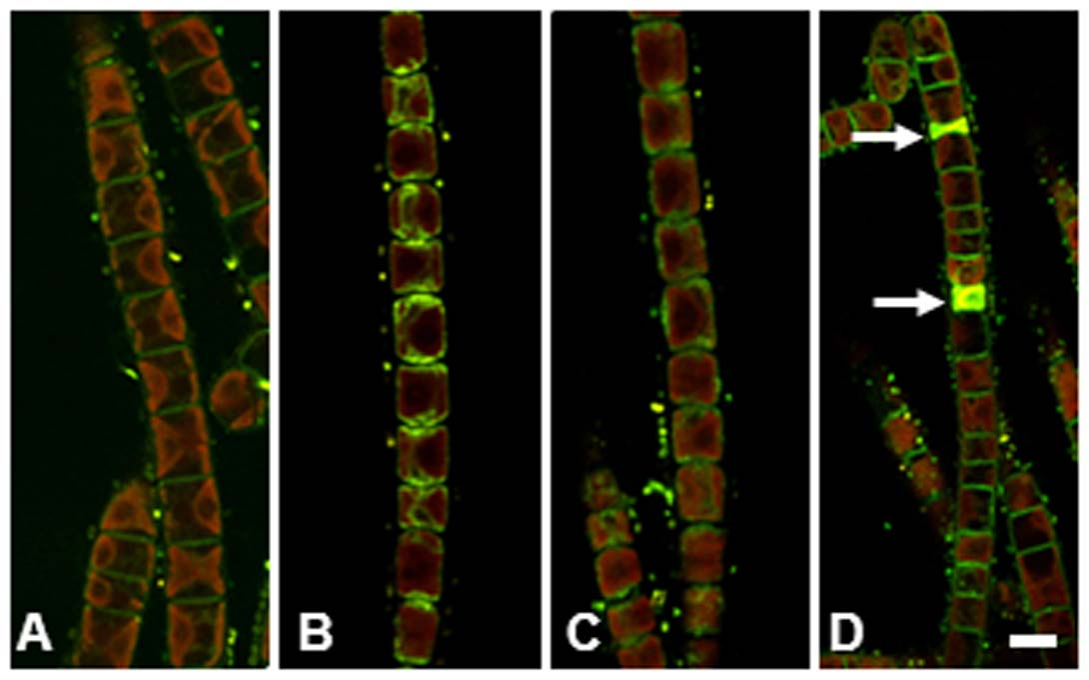
*Klebsormidium crenulatum* stained with FM 1–43. (A) Control cells. (B) Desiccated for 2.5 h. (C) 2 h recovery in culture medium after drying for 2.5 h. (D) Control filament with two dead cells (arrows). Bar 10 µm.

To assess the cellular damage resulting from the desiccation stress, a cell viability test using the FM1-43 dye was performed. In control cells of *K. crenulatum*, FM1-43 dye stained the cell membrane predominantly (Fig. 2 A); however in some cases, the membranes of the vacuoles and chloroplast were also stained if the cells were exposed to the dye for a longer period (not shown). In contrast, dead cells, which are always present in small numbers in a cell culture, showed a bright fluorescence in the cell lumen (Fig. 2 D).

The cytoplasm of cells exposed to desiccation stress for 2.5 h was stained in a granular pattern; however, the plasma membrane was still visible and no labeling of the plastid was observed (Fig. 2 B). After a recovery period of ∼2 h the plasma membrane was stained more prominently as in the desiccated cells, although weaker than in the control cells, and there was still some cytoplasmic staining (Fig. 2 C). However, we rarely observed the bright fluorescence of the total cell volume, indicative of dead cells (Fig. 2 D).

### Molecular analysis

To identify desiccation stress-regulated cellular processes and functions in *K. crenulatum,* we first established a reference transcriptome database. Algae were transferred to filter membranes and RNA was isolated either directly or after applying desiccation stress (2.5 h desiccation over silica gel). RNA was isolated from control and desiccated samples (each in triplicate). Control samples contained more RNA than desiccated samples (Fig. S2, compare the amount of 18S and 25S between control and desiccated samples), indicating a considerable reduction in the amount of RNA during desiccation stress in *K. crenulatum*. In addition, in the electropherograms of the desiccated samples, the 18S and 25S rRNA peaks were reduced and an increased baseline noise and lower RNA integrity number (RIN between 5.7 to 5.8 in the control samples and between 3.8 to 4.2 in the desiccated samples) were observed, indicative of RNA degradation, which is nearly absent from the control samples (see also discussion).

For the reference library, all samples were pooled (same amount of RNA from each sample), and a normalized random primed cDNA library was prepared and sequenced using 454 sequencing. Random primed cDNA libraries were also prepared from the individual samples and sequenced using the Illumina technology. All control samples and two desiccated samples yielded sufficient reads for further analyses. In the following sections, we will first describe the reference library and then present our results from the expression analysis.

### Reference library

454 sequencing yielded 1,553,953 sequenced reads (average read length 409 b, GC-content 52.17%, N 0.02%), giving more than 138,213 contigs with a mean length of 563 b. However, 83,276 of the contigs represented short singletons; therefore a high-coverage reference database was constructed, which contained only contigs containing at least 5 reads. This database contained 24,183 contigs with a mean sequence length of 1,327 b (N50 = 1,463 b). All further analyses were performed using this high-coverage assembly.

#### BLAST-analyses

To identify transcripts of interest, we compared the deduced peptides of the high-coverage assembly with the proteomes of selected green algae and embryophytes (Table 1) using BLASTX. A total of 13,316 contigs (55.1%) showed similarity to the deduced protein sequences in the genomes (for simplicity, henceforth called proteins) of the selected viridiplants (chlorophytes and streptophytes, e-value <exp -10). 10,505 *K. crenulatum* contigs = 43.4% showed similarity to chlorophyte protein sequences, whereas 12,596 K. *crenulatum* contigs = 52.1% showed similarity to streptophyte protein sequences. However, only 4,752, 5,949 and 7,701 *K. crenulatum* contigs showed similarity to proteins in all investigated viridiplants, chlorophytes or streptophytes respectively. Overall, more sequences showed similarity to streptophyte genomes than to chlorophyte genomes (Table 1) and generally the conservation of the amino acid sequences was higher when the deduced peptide sequences of *Klebsormidium* contigs were compared with streptophyte proteins than when comparing with chlorophyte proteins (not shown). These results highlight the genetic diversity of chlorophytes and the large number of streptophyte-specific contigs (2,791 *K. crenulatum* contigs).

**Table 1:** Similarity of *Klebsormidium crenulatum* contigs to other organisms.

Name	Growth type	Number of Blast hits	Up-regulated	Up-regulated (%)
*Chlamydomonas reinhardtii*	Unicellular Flagellate	8727	1545	17.70
*Coccomyxa subellipsoidea*	Unicellular Coccoid	8856	1624	18.34
*Micromonas pusilla*	Unicellular Flagellate	7759	1367	17.62
*Ostreococcus lucimarinus*	Unicellular Coccoid	7180	1273	17.73
*Physcomitrella patens*	Moss	11842	2095	17.69
*Selaginella italica*	Lycophyte	10781	1886	17.49
*Zea mays*	Grass	10771	1884	17.49
*Oryza sativa*	Grass	10590	1879	17.74
*Aquilegia coerulea*	Perennial Herb	10995	1924	17.51
*Solanum tuberosum*	Perennial Herb	10127	1870	18.47
*Eucalyptus grandis*	Tree	10897	1875	17.21
*Theobroma cacao*	Tree	11137	1945	17.46
*Arabidopsis thaliana*	Annual Herb	10995	1931	17.56
*Malus domesticus*	Tree	10670	1890	17.71
*Medicago trunculata*	Annual Herb	9552	1742	18.24
*Populus trichocarpa*	Tree	11070	1931	17.44
*Ricinus communis*	Shrub	11015	1907	17.31

24,183 contigs were blasted against the protein database of the selected organisms listed (obtained from Phytozome). The number of BLASTX hits (e-value <exp -10) is shown. The number of hits of contigs that were up-regulated under desiccation stress (twofold or more) and the percentage of the total hits are also shown.

#### KEGG-Modules and Pathway analyses

To investigate whether the reference genome covers most cellular functions, we searched the KEGG databases using the high-coverage contigs as a query. As shown in Table S1, most basic cellular processes (e.g. carbohydrate metabolism, fatty acid metabolism, nucleotide metabolism, amino acid metabolisms, photosynthesis, and respiration) were completely or nearly completely represented in the deduced peptide sequences.

### Expression analysis

Illumina sequencing of random primed cDNA libraries yielded between 12 and 30 million reads per sample. The read statistics are given in Table S2. Only high-quality reads (unique or duplicates) were used for further analyses. Table 2 gives an overview of the number of *K. crenulatum* contigs showing changes in transcript expression level. 10,493 *K. crenulatum* contigs (43.4%) showed at least a 2-fold change in their expression level, thus the majority of the transcriptome is apparently unaffected. Only a few K. *crenulatum* contigs were expressed exclusively under desiccation stress or control conditions (Table 2) and more transcripts were down-regulated than up-regulated (Table 2). Interestingly, the percentage of up-regulated transcripts showing similarity to viridiplant genomes is higher than the percentage of down-regulated transcripts showing similarity to viridiplant genomes. In Table 3 the 20 highest up-regulated *K. crenulatum* contigs are shown. The complete lists of all up- and down-regulated mRNAs including the most similar proteins in the 14 viridiplant proteomes searched, are presented in Table S3 and Table S4. Most of the highly up-regulated contigs do not show similarity to known viridiplant proteins and many show no similarity to any known protein at all. Thus a large number of regulated transcripts might represent specific adaptations of *K. crenulatum* to its environment. However, 100 of the 171 *K. crenulatum* contigs, up-regulated at least 10-fold, show similarity to proteins in viridiplant proteomes. Surprisingly, we could detect only 46 *Arabidopsis* proteins in this category. Interestingly, most of these *Arabidopsis* proteins are well conserved in embryophytes and 34 are also similar to proteins in chlorophytes, suggesting that these proteins represent basic cellular functions, generally up-regulated upon stress. In contrast, we could detect several additional *K. crenulatum* contigs (at least 10-fold up-regulated) that show similarity to other embryophytes. Overall there are 89 *K. crenulatum* contigs similar to proteins in *Medicago* and 61 to 71 *K. crenulatum* contigs similar to proteins from the trees investigated (except *Eucalyptus*, 49).

**Table 2:** Overview of changes in transcript expression during desiccation stress.

	Up-regulated	Down-regulated
At least 2-fold change	4129 (2264)	5898 (2343)
•Significant (P_val_ * <0.05):	3766	5011
•Significant (P_adj_ * <0.05):	3642	4584
•Number of KO-Terms	736	616
Expressed only in/upon controls/desiccation	31	435
•Significant (P_val_ * <0.05):	4	136
•Significant (P_adj_ * <0.05):	2	115

The number of *Klebsormidium crenulatum* contigs in each category is given except for Number of KO-terms, which gives the number of KO-Terms, obtained for up- and down-regulated *K. crenulatum* contigs with a P_adj_ <0.05. Number in brackets gives the percentage of *K. crenulatum* contigs that show similarity to viridiplant genomes.

*obtained with the DeSeq program. P_val_  =  p value for the statistical significance of this change.

P_adj_  =  p value adjusted for multiple testing with the Benjamini-Hochberg procedure, which controls the false discovery rate (FDR).

**Table 3:** *Klebsormidium crenulatum* contigs showing the largest changes upon desiccation stress.

K. crenulatum -ID	Fold Change	padj	Function
UN027167	Not expressed in controls	0.03141657	Plastid NADH:ubiquinone/plastoquinone oxidoreductase
UN034964	Not expressed in controls	0.00627352	
UN022359	91.4439431	0.00021721	
UN036634	60.5266278	8.9783E-16	Similar protein in some plants
UN043434	52.011157	7.675E-16	
UN036811	51.8868523	1.6101E-23	Similar protein in some plants
UN012067	51.0430016	1.3086E-11	Similar protein in some plants
UN034707	47.3497452	2.6678E-17	Similar protein in some plants
UN020446	45.7941202	1.3302E-21	Similar protein in some plants
UN000210	38.6089069	7.6031E-16	
UN026331	38.1383995	1.679E-07	Similar protein in some plants
UN028420	35.9012593	4.4486E-83	ELIP1 early light inducible protein
UN031973	35.1401051	1.6143E-65	NPQ4 (PSBS)
UN022671	33.789896	7.0903E-37	
UN035517	30.9168329	5.324E-104	Similar protein in some plants
UN052587	30.7842929	6.0114E-36	
UN028622	30.4695717	1.9817E-51	PHT6 phosphate transporter
UN026579	30.0812794	1.0935E-69	GLT1 plastid glucose transporter
UN030270	29.7741941	5.6407E-34	
UN029908	29.432577	2.1169E-81	Catalase 2

A tentative function is given when possible.

#### KEGG analysis of desiccation induced changes

**Figure 3:**
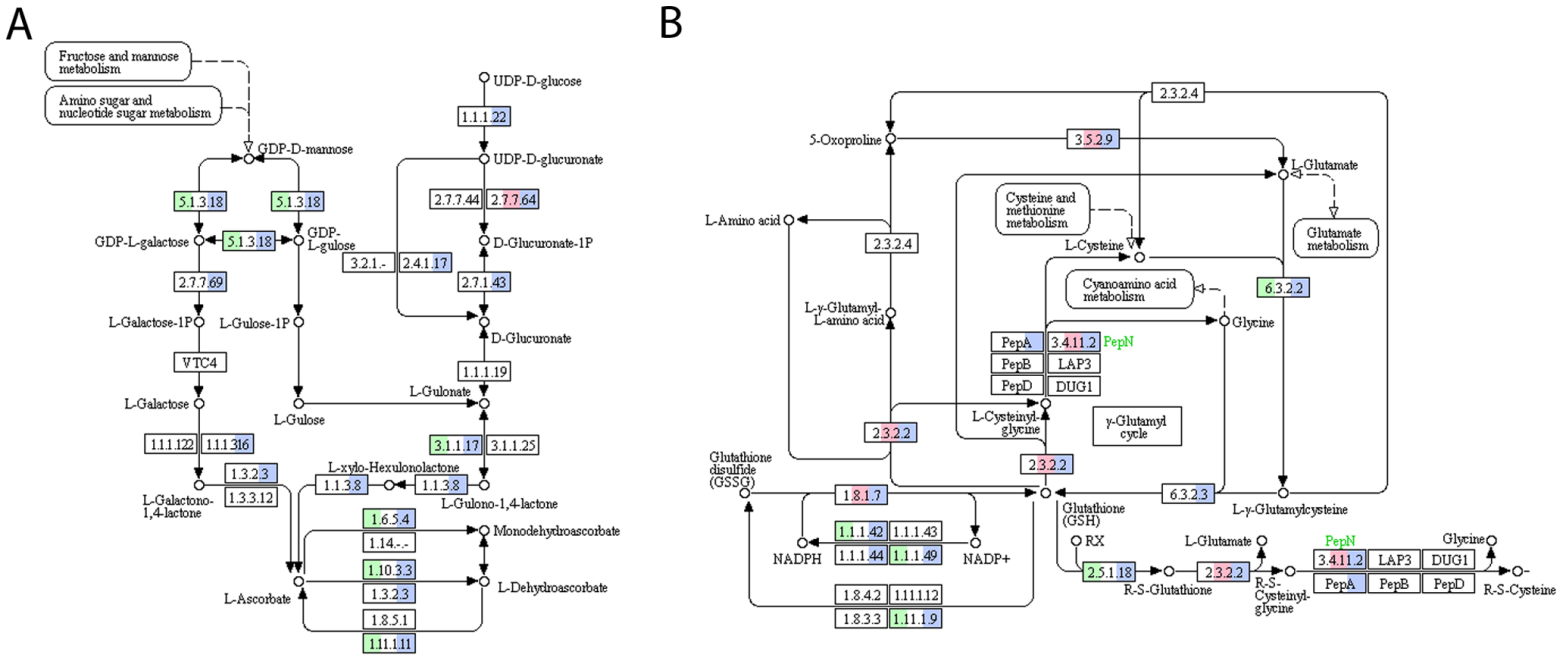
Ascorbate (A) and glutathione (B) metabolism in *Klebsormidium crenulatum*. (A) Biosynthesis of ascorbate via the D-galacturonate pathway is upregulated, as are ascorbate-consuming and recycling enzymes. (B) The biosynthesis of glutathione is up-regulated except for glutathione synthase. Glutathione degradation is down-regulated, whereas glutathione-consuming enzymes are up-regulated.

**Figure 4:**
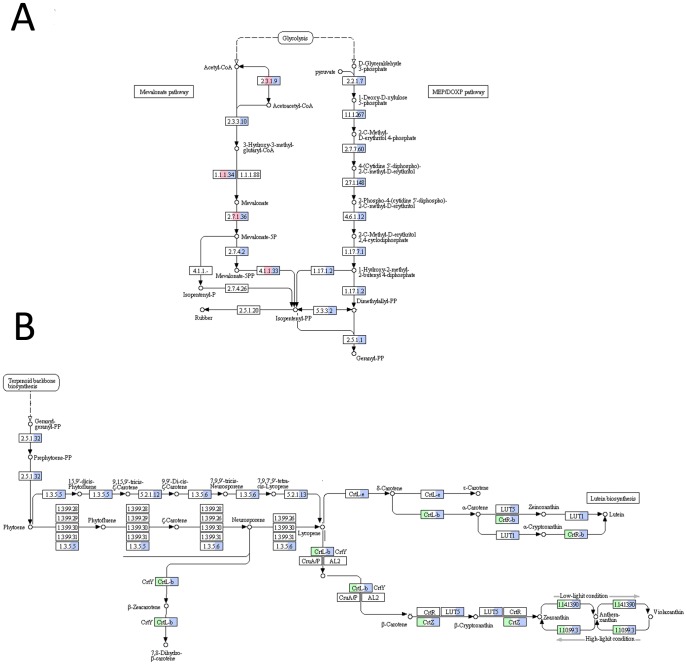
Biosynthesis of carotenoids in *Klebsormidium crenulatum*. (A) Terpenoid backbone synthesis, the cytosolic mevalonate pathway is inhibited. (B) Carotenoid biosynthesis is up-regulated.

To gain more insight into the molecular mechanisms of desiccation tolerance, we searched the KEGG database for pathways (Table 4) and modules (Table S1), using the complete, the up-regulated and the down-regulated datasets. *K. crenulatum* contigs could be assigned to 782 different KO functions using the pathway searching tool, and transcripts representing 229 KO functions were up-regulated and transcripts representing 186 KO functions were down-regulated, respectively. The majority of the deduced KEGG-pathways (for simplicity called pathways in the following sections) showed complex regulation patterns with several contigs up- and down-regulated (e.g. Figs. 3 and 4). The 15 pathways which were overall most strongly up- and down-regulated are shown in Table 4 and Table 5. As is evident from Table 4, contigs involved in energy production (glycolysis, TCA cycle, respiration, photosynthesis etc.) were mainly up-regulated. Other important up-regulated pathways were galactose, ascorbate (Fig. 3 A) and glutathione (Fig. 3 B) metabolism; and plant hormone and calcium signaling. No contigs showed similarity to proteins of the auxin and gibberellin signal transduction pathways. In contrast, homologues for all components of the cytokinin signaling pathway (CRE1, down-regulated; AHP, up-regulated; B-ARR, down-regulated and A-AAR, up-regulated) and 3 of the 4 components of the abscisic acid signaling pathway (PP2C, SnRK2, ABF, all up-regulated) were found. Surprisingly, putative homologues of the ethylene and jasmonic acid receptors (ETR, JAR1) are also present. However, putative homologues of other components of these signaling pathways are missing, except for (CTR1 and EIN3) for which we also detected putative homologues.

**Table 4:** Effect of desiccation stress on transcript expression in *Klebsormidium crenulatum*: strongly up-regulated pathways.

KO number	Cellular Function	All contigs	Up-regulated contigs	% up-regulated	Down-regulated contigs	% down-regulated
ko00195	Photosynthesis	32	30	93.8	1	3.1
ko03010	Ribosome	124	114	91.9	4	3.2
ko00196	Photosynthesis - antenna proteins	12	9	75.0	1	8.3
ko00710	Carbon fixation in photosynthetic organisms	21	14	66.7	2	9.5
ko00190	Oxidative phosphorylation	79	48	60.8	5	6.3
ko04075	Plant hormone signal transduction	14	8	57.1	5	35.7
ko00030	Pentose phosphate pathway	18	10	55.6	4	22.2
ko00020	Citrate cycle (TCA cycle)	20	11	55.0	1	5.0
ko00630	Glyoxylate and dicarboxylate metabolism	29	15	51.7	4	13.8
ko00052	Galactose metabolism	15	7	46.7	4	26.7
ko00071	Fatty acid degradation	13	6	46.2	4	30.8
ko04020	Calcium signaling pathway	11	5	45.5	3	27.3
ko00010	Glycolysis/Gluconeogenesis	32	14	43.8	8	25.0
ko00053	Ascorbate and aldarate metabolism	15	6	40.0	2	13.3
ko00500	Starch and sucrose metabolism	36	14	38.9	15	41.7

The KEGG ontology (KO) numbers were retrieved using the KAAS annotation server. Using the KO numbers, pathway maps were constructed using the KEGG pathway reconstruction tool. The pathways with the largest percentage of up-regulated KO functions are shown. Human disease-related pathways and overview maps are not shown.

**Table 5:** Effect of desiccation stress on transcript expression in *Klebsormidium crenulatum*: strongly down-regulated pathways.

KO number	Function	All contigs	Up-regulated contigs	% up-regulated	Down-regulated contigs	% down-regulated
ko03450	Non-homologous end joining	9	2	22.2	6	66.7
ko04140	Regulation of autophagy	11	1	9.1	7	63.6
ko00970	Aminoacyl-tRNA biosynthesis	27	3	11.1	15	55.6
ko03420	Nucleotide excision repair	35	5	14.3	19	54.3
ko03030	DNA replication	28	1	3.6	15	53.6
Ko03430	Mismatch repair	19	1	5.3	10	52.6
ko04142	Lysosome	43	7	16.3	21	48.8
ko00310	Lysine degradation	19	5	26.3	9	47.4
ko04150	mTOR signaling pathway	13	3	23.1	6	46.2
ko04111	Cell cycle - yeast	48	6	12.5	22	45.8
Ko00670	One carbon pool by folate	11	1	9.1	5	45.5
ko00513	Various types of N-glycan biosynthesis	20	2	10.0	9	45.0
ko04113	Meiosis - yeast	38	3	7.9	17	44.7
ko00640	Propanoate metabolism	23	6	26.1	10	43.5
ko00910	Nitrogen metabolism	14	4	28.6	6	42.9

The KEGG ontology (KO) numbers were retrieved using the KAAS annotation server. Using the KO numbers, pathway maps were constructed using the KEGG pathway reconstruction tool. The pathways with the largest percentage of down-regulated KO functions are shown. Human disease-related pathways and overview maps are not shown.

#### Desiccation induced up-regulation of transcripts

Only for six of the top 20 up-regulated contigs it is possible to infer a putative function. Four of these transcripts are similar to plastidic proteins: two up-regulated transcripts are possibly involved in non-photochemical quenching (UN028420, ELIP1, 35.9-fold up-regulated; UN031973, NPQ4 (PSBS), 35.1-fold up-regulated); another interesting contig shows similarity to a plastidic glucose transporter (UN026579, GLT1, 30.1-fold up-regulated). Transcripts for sucrose phosphate synthase (2 contigs: UN036592, 3.6-fold; UN039619, 2.8-fold) and sucrose synthase (3 contigs: UN023940, 2.1-fold, UN038702 3.2-fold; UN039004 3.8-fold) are also up-regulated, whereas transcripts for cellulose synthase and some enzymes of the starch and trehalose metabolism are down-regulated. We also found a contig displaying strong similarity to AT2G35840, a sucrose-6-phosphate phosphohydrolase family protein (e-value: 3e-104). This transcript is 4-fold up-regulated; however, the pathway reconstruction tool at KEGG failed to recognize this transcript as a putative SPSP.

**Figure 5:**
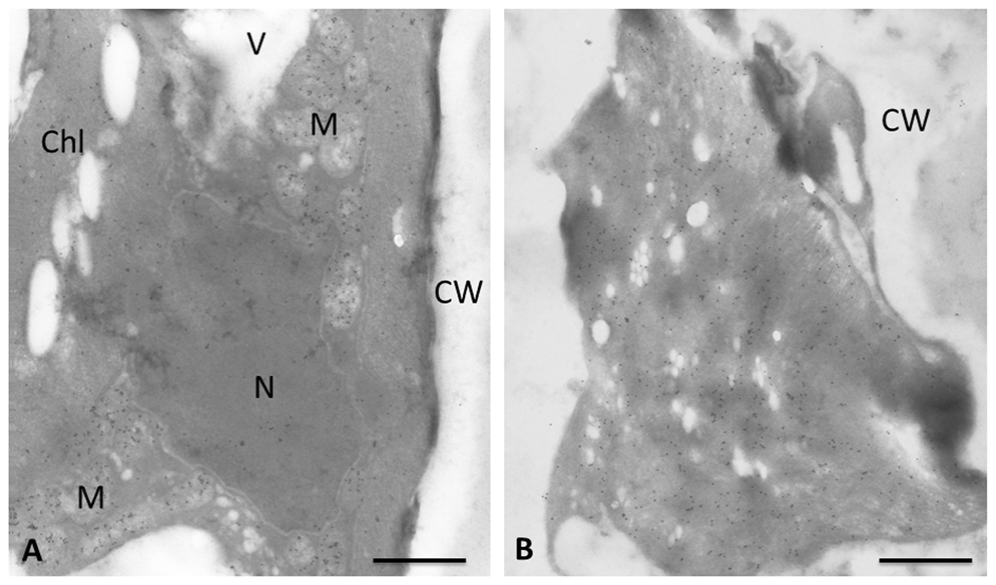
Immunogold labeling with 10 nm gold particles, of glutathione in transmission electron microscopic sections of *Klebsormidium crenulatum*. (A) Control cell. (B) Desiccated cell (2.5 h at ∼ 10% RH). Bar 1 µm.

**Figure 6:**
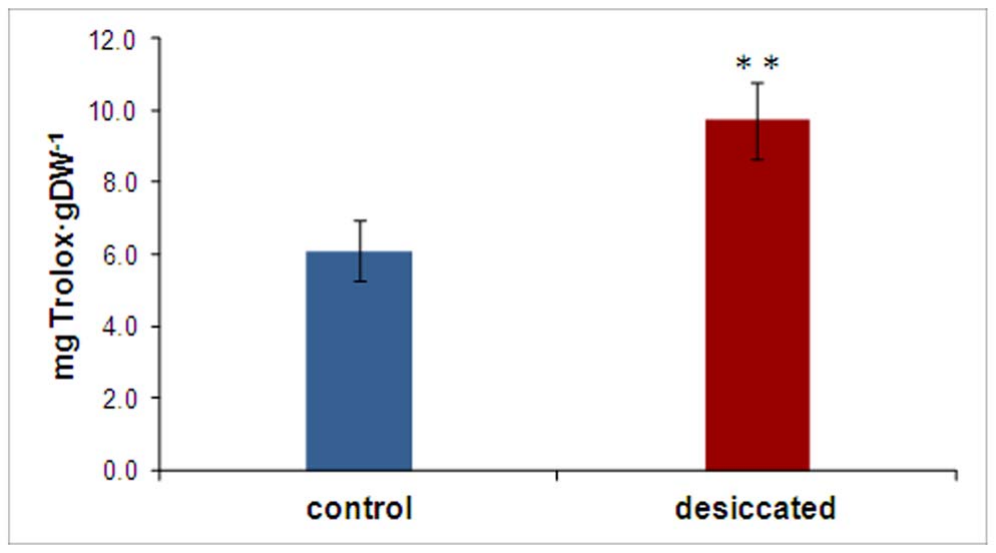
Antiradical power (Trolox equivalents g^−1^ dry weight (DW) of *Klebsormidium crenulatum* control samples (n = 3) and samples (n = 3) desiccated above silica gel for 2.5 h. The values are expressed in mg Trolox equivalent.g dry weight (DW)^−1^. Means and standard deviations are shown. The statistical siginficance was thested by two sample *t*-test and demonstrated that the antiradical power of the desiccated samples is significantly higher (p <0.01).

Several transcripts for putative enzymes of the galactinol/raffinose metabolism were found to be up-regulated, including a transcript for galactinol synthase (UN010053, BLASTX result: AT1G60450.1, AtGolS7, e-value 5 exp-30), the key enzyme for the biosynthesis of the raffinose family of oligosaccharides (RFO). Ascorbate is probably synthesized from GDP-Man as a transcript similar to GDP-D-mannose 3′, 5′-epimerase (UN025544, similar to AT5G28840.2, GME, e-value: 3E-49) is under desiccation stress 26-fold up-regulated, as are several contigs with similarity to enzymes using ascorbate as reductant. Similarly to ascorbate, glutathion plays an important role in reactive oxygen metabolism. Several transcripts coding for enzymes in the glutathion metabolism are up-regulated (Fig. 3 B). For example, contigs with similarity to enzymes involved in the first step of glutathion biosynthesis, glutathione-S-transferase and glutathione peroxidase are up-regulated, while transcripts with similarity to glutathione-degrading enzymes and the glutathione-disulfide reductase are down-regulated. The importance of reactive oxygen species (ROS) protection is also highlighted by the up-regulation of a transcript similar to catalase 2 (UN029908, 29.4-fold up-regulation). To corroborate these findings, we investigated changes in the total cellular glutathione content, using an immune gold glutathione-labeling approach (Fig. 5), and the cellular antiradical power of *K. crenulatum*. The distribution of glutathione-specific gold labeling in *K. crenulatum* grown under control conditions revealed that the highest glutathione labeling density was found in mitochondria, followed by nuclei, chloroplasts and the cytosol (Fig. 5 A and Table 6). Due to ultrastructural changes induced by desiccation, gold particles could not be clearly assigned to the individual cell compartments of desiccation-stressed *K. crenulatum* cells (Fig. 5 B). Thus, the total amount of glutathione labeling was calculated for both control and desiccation-stressed *K. crenulatum* cells (Table 7). The glutathione labeling density increased in desiccation-stressed *K. crenulatum* cells to about 177% of the value in control cells (Table 6). The total cellular antiradical power was determined using the DPPH-test (see material and methods). The cellular antiradical power was significantly higher (p <0.01) in 2.5 h desiccated samples than in control cells (Fig. 6).

**Table 6:** Glutathione content of *Klebsormidium crenulatum* cells.

	Gold particles per μm^2^	
	control	desiccated
Mitochondria	168±8^a^	n.d.
Chloroplasts	28±1^d^	n.d.
Nuclei	42±2^ c^	n.d.
Cytosol	29±1^d^	n.d.
Whole cell	30±1^e^	53±2^b^
Cell without vacuole	32±1^e^	n.d.

Values are means with standard errors, and document the total amount of gold particles bound to glutathione per μm^2^ in different cell compartments and whole cells of *Klebsormidium crenulatum* grown under control and desiccation conditions. n = 40 for the individual cell structures and n = 25 for the whole cell. Different lowercase letters indicate significant differences (P<0.05) analyzed with the Kruskal-Wallis test followed by post-hoc comparison according to Conover. n.d. = not determined.

**Table 7:** Plant desiccation/dehydration/drought stress-related contigs in *Klebsormidium crenulatum*.

Name	*Arabidopsis* Gene ID	Up/down-regulation	Padj	Similarity to the *Ath* gene
*Late embryogenesis abundant (LEA) proteins*				
LEA-related	AT5G60530.1	0.23991374	2.5624E-05	6E-18
**LEA_2**	**AT2G44060.2**	**1.86413136**	**0.00014204**	**1E-60**
LEA-related	AT1G54890.1	0.29400396	6.7547E-07	5E-13
LEA-related	AT5G54370.1	1.22790739	0.53229844	4E-16
**LEA_4**	**AT5G44310.2**	**3.76509689**	**1.5892E-16**	**1E-11**
**LEA-related Hydroxyproline-rich glycoprotein**	**AT3G44380.1**	**4.68751795**	**1.8879E-16**	**1E-17**
**LEA_4**	**AT5G44310.2**	**4.25267018**	**1.2762E-18**	**4E-13**
**LEA_4**	**AT5G44310.2**	**4.25267018**	**1.2762E-18**	**4E-13**
**LEA-related**	**AT3G62580.1**	**3.00106737**	**8.899E-13**	**8E-41**
**LEA-related Hydroxyproline-rich glycoprotein**	**AT2G01080.1**	**3.882294126**	**0.00435072**	**3E-11**
**LEA-related Hydroxyproline-rich glycoprotein**	**AT2G01080.1**	**4.659043737**	**4.6237E-05**	**2e-11**
LEA-related	AT3G62580.1	3.993800234	0.39220796	4E-17
*Early responsive to dehydration stress (ERD) genes*				
ERD-family protein	AT4G22120.6	0.40330981	0.00264642	eE-35
**ERD-family PM protein**	**AT4G04340.1**	**1.914357304**	**0.00119979**	**3E-29**
ERD-family PM protein	AT4G04340.1	0.719668846	0.54074033	4E-16
**ERD4**	**AT1G30360.1**	**3.326696704**	**0.09599311**	**3E-46**
**ERD4**	**AT1G30360.1**	**2.282664946**	**0.00333271**	**1E-148**
**ERD4**	**AT1G30360.1**	**4.305228158**	**7.4444E-15**	**4E-22**
**ERD4**	**AT1G30360.1**	**4.069239817**	**3.643E-21**	**1E-169**
**ERD4**	**AT1G30360.1**	**2.682142777**	**0.54503736**	**5E-61**
ERD-family PM protein	AT4G04340.1	1.200513952	0.40890632	1E-121
ERD4	AT1G30360.1	0.347378285	1.6875E-06	5E-166
ERD-family protein, localized in endomembrane system	AT4G02900.1	0.851420448	0.49554025	1E-100
*Other*				
**Chloroplast drought-induced stress protein of 23 kDa, CIDSP32, thioredoxin**	**AT1G76080.1**	**1.71514379**	**0.00189019**	**5E-74**
Drought-sensitive 1, DRS1,	AT1G80710.1	1.574535846	0.17300482	3E-64
**Drought-sensitive 1, DRS1,**	**AT1G80710.1**	**1.722093198**	**0.03356124**	**3e-63**

Significantly up-regulated genes are in bold.

#### Desiccation induced down-regulation of transcripts and complex regulation patterns

Pathways, which were strongly down-regulated, were mainly involved in integrative cellular functions, such as cell division and DNA repair mechanisms. Purine-, pyrimidine-, most cofactors, and many amino acid biosyntheses were down-regulated as well. However, as mentioned above, most pathways showed complex regulation patterns. Fig. 4 illustrates this for the terpenoid backbone (Fig. 4 A) and carotenoid biosynthesis (Fig. 4 B). Contigs for the cytosolic mevalonate pathway are down-regulated, but contigs for the plastidic MEP/DOXP-pathway are not regulated, and many contigs of the carotenoid pathways are up-regulated and none down-regulated. In agreement with this, we also observed up-regulation of contigs coding for most light-harvesting complex proteins psbS and elip (see above), supporting the idea that non-photochemical quenching plays an important role in protection from ROS during desiccation stress.

Protein degradation using the ubiquitin/proteasome system is another interesting example with a complex regulation pattern; contigs for all core subunits are up-regulated, as well as some contigs for the enzymes of the ubiquitination mechanism. However, transcripts for some other components of the ubiquitination mechanism and some components of the regulatory proteasome lid and were down-regulated. In protein synthesis, contigs for nearly all ribosomal proteins were up-regulated, while contigs for some components of the nuclear ribosome processing pathway and some aminoacyl-tRNA synthases were down-regulated.

#### Embryophyte desiccation/dehydration/drought stress-related proteins

Several *K. crenulatum* contigs showed similarity to embryophyte proteins implicated in the desiccation response (Table 7). Generally, more than one contig showed similarity to a certain *Arabidopsis* gene, suggesting the presence of small protein families. Most contigs were significantly up-regulated, however a few contigs were not-regulated or down-regulated.

## Discussion

In the present study we investigated the transcriptome and physiology of *K. crenulatum* under strong desiccation stress, including tests of cell viability. Cell viability in *K. crenulatum* was previously tested by using the amphiphilic styril dye FM 1–43 [17]. The present study corroborated the previous results, as we found the plasma membranes stained in control cells, whereas in desiccated cells, only slight uptake of the dye was observed. FM dyes only fluorescence under hydrophobic conditions, and mechanisms of uptake into embryophyte plant cells have been discussed [47], [48]. However, even under the strong desiccation conditions used, the total cell volume was not stained, suggesting that the damage was not as extensive as reported in cells that were desiccated for 7 days [17]. In contrast, initially dead cells of control filaments show a bright FM 1–43 fluorescence throughout the whole cell lumen. We cannot fully exclude the presence of damage to the cell membrane or changes in the cell membrane composition caused by our desiccation treatment; however, these results indicate that cells survive strong desiccation over silica gel. To further analyze the physiological state of the desiccated *K. crenulatum* cells, photosynthetic parameters were measured. The CO_2_ assimilation rate of *K. crenulatum* cells was determined for the first time in the present study. By gas exchange measurements it is possible to determine CO_2_ assimilation rates of *K. crenulatum* under defined RH leading to desiccation under controlled conditions (temperature, CO_2_ and H_2_O content, light). Until now most measurements of photosynthetic parameters in green algae were performed in the fluid phase. Therefore, so far only chlorophyll fluorescence parameters could be determined in the desiccated state (e.g. [3]), but no information on assimilation rates was available.

During the desiccation period of 2.5 h under low light conditions (30 µmol photons) the assimilation rate dropped below zero, indicating that the cells shut down their photosynthetic activity; the still-measurable respiration rate might be attributed to failure to reach equilibrium to 20% RH in the chamber of the GFS-3000 (it is not possible to reach RHs as low as obtained in the desiccation box over silica gel); however after 21 h recovery, both the assimilation rate and Yield II were near zero, indicating drastic perturbation of the cells. Suppression of photosynthesis in green algae during dehydration has been previously described [13], [16], [19], [49]. In an earlier study using *K. crenulatum,* short-term desiccation for 3 hours at ambient room temperature and humidity was performed [16]. There, the F_v_/F_m_ value recovered after 30 min rehydration to 48% of the initial value, and after 2 h the cells were fully recovered [16]. In a study using *K. dissectum* desiccated under different RH (100, 55 and 5%) for 1 or 3 weeks, the cells recovered within 1 or 2 weeks respectively, indicating that some cells have the ability to survive these harsh treatments [19].

### Transcriptome analysis

High-quality RNA is crucial for any transcriptome analysis. RNA agarose gels and Bioanalyzer electropherogram profiles indicated a reduced amount of RNA and some RNA degradation in our desiccated samples, but little or no degradation in our control samples (Fig. S2). We are convinced that the reduced RNA amount and decreased RNA integrity number are not experimental artifacts, but rather were caused by the applied stress conditions, for the following reasons: (1) The same protocol was used for all samples and samples were processed in parallel. (2) Similar results have been obtained for other systems, e.g. *Tortula ruralis* gametophytes [50], pea seeds [51] and yeast [52]. Particularly in the study by Chen et al. [51], a detailed analysis of the RNA integrity numbers (RIN, [53]) is given, and similar values to those obtained in this study were found in artificially aged pea seeds, which were considered as ‘partially degraded’ but still usable for the analysis.

To our knowledge this is the first broad-scale expression study of a *Klebsormidium* species in response to abiotic stress. ESTs have been sequenced for *Klebsormidium flaccidum* and *Klebsormidium subtile*, yielding about 60,000 ESTs (NCBI, 22.07.2014). As no reference data were available for *K. crenulatum*, we first established a reference transcriptome based on the pooled RNA samples. This reference transcriptome contains over 24,000 contigs. About 60% are similar to proteins in sequenced viridiplant genomes, with about 10% apparently present only in streptophytes, supporting the close relationship of *Klebsormidium* with embryophytes. However, currently the number of genomes from chlorophytes is limited. Therefore it seems possible that the addition of further genomes will increase the total number of *K. crenulatum* contigs that are similar to chlorophyte proteins. The established reference transcriptome covers all major cellular functions and metabolic pathways. However, for more than 30% of the contigs, no similar protein was found in any database, suggesting the existence of a large number of genus- or species-specific genes, which is very similar to the situation observed in other organisms [54].

### Expression Analysis

Our expression analysis revealed that a large number of contigs are up- or down-regulated. Given the large number of contigs that show no similarity to any organism and which are strongly up-regulated in *K. crenulatum,* we did not perform any GO or KEGG-ontology enrichment study, but instead we focused on KEGG pathway analyses. There are three major responses to desiccation in *K. crenulatum*. First, contigs related to energy metabolism (glycolysis, TCA cycle, respiration) are up-regulated. The role of these pathways during desiccation tolerance is not clear. In actively growing roots and whole plants water stress inhibits mitochondrial respiration; however, in leaves the response is more variable, ranging from reduction in most cases over no change even to increase under severe water stress e.g. in *Arnica alpina*, *Triticum aestivum* or *Helianthus annuus* [55]. As the algal cell is more comparable with a plant leaf than with plant roots or seeds, we suggest that similar to the above mentioned examples, *Klebsormidium* cells try to increase respiration for energy production, which might also help to explain the high respiration rate in the desiccated state, however it seems also possible that the cells just prepare for a fast energy production upon rehydration.

Photosynthesis is inhibited very rapidly upon desiccation (see above). *Klebsormidium* responds by up-regulation of genes involved in photosynthesis, obviously preparing for rehydration. Finally, the cells show strong protection against light damage and ROS stress, by increasing carotenoid and LHC biosynthesis as well as up-regulating glutathione and ascorbic-acid metabolism. The observed up-regulation of the pentose-phosphate cycle is probably required to provide the cells with the reducing agent NADPH. Overall these responses are very similar to typical embryophyte responses [56]. Interestingly, we found in *K. crenulatum* contigs that are similar to well-known plant proteins involved in the desiccation/drought response, e.g. LEA proteins [57] and genes involved in early response to drought [58] further emphasizing the similarities between the streptophyte algal and plant desiccation responses.

### Osmolytes

It has been previously reported that in *Klebsormidium flaccidum,* sucrose is increased upon cold stress [59], and a similar reaction could be expected after desiccation stress. Indeed, we observed an up-regulation of sucrose synthase (K00695) and sucrose phosphate synthase (K00696). Nagao and Uemura [60] found that sucrose-phosphate phosphatase is present in *K. flaccidum*, but has a different structure, lacking an extensive C-terminal domain. In agreement with this, we observed a transcript similar to a plant sucrose-phosphate-phosphohydrolase family protein, which could not be mapped on the sucrose pathway by the reconstruct-pathway tool at KEGG. In addition to sucrose, members of the raffinose family of oligosaccharides (RFO) have been shown to be compatible solutes involved in tolerance of stresses such as desiccation and cold in plants [61]. Previously raffinose has been detected in *K. crenulatum* as one of the major soluble carbohydrates [18]. Contigs for several enzymes of the galactinol/raffinose metabolism were up-regulated in desiccated samples, suggesting that RFOs might function as compatible solutes in *K. crenulatum* as well.

### Phytohormones

Several phytohormones have been implicated in signaling abiotic stress responses (recently reviewed [62]). ABA has been implicated in signaling of cold and desiccation responses [63] and we could detect contigs similar to all components of the ABA signaling pathway except the ABA receptor (PYR/PYL) in *K. crenulatum,* suggesting that this pathway might have evolved in streptophyte algae and facilitated colonization of the terrestrial habitat. It has been suggested that jasmonic acid acts even before ABA in the stress response [64]. Interestingly, a contig similar to JAR1 (the embryophyte jasmonic acid receptor) was also found, but no other contig showed similarity to other components of the jasmonic acid signaling pathway. This raises the possibility that the jasmonic acid receptor might function as an ABA receptor in *K. crenulatum*. Further work will be required to clarify this possibility.

### Lipids

Air drying has recently been shown to increase the synthesis of triacylglycerol in the green alga *Chlorella kessleri* [65]. The authors subjected their samples to ambient air conditions on glass filters for 11 h, and obtained an increase of triacylglycerols from 0.3 to 15.3% (w/w), which corresponds to an increase from 4.7 to 70.3 mole% of fatty acids; the cell weight increased 2.7-fold in 96 h [65]. Our data also suggest that an increase in fatty acid production can be expected from the up-regulation of the respective enzymes. However, for acquiring desiccation tolerance, the composition of the biomembranes was found to be more crucial, as they are the initial targets in the desiccation process [66]. Moreover, chloroplast membranes are protected, as these authors reported the removal of the thylakoid lipid monogalactosyldiacylglycerol (MGDG), which was hydrolyzed and converted into diacylglycerol (DAG). It was then further converted into phosphatidylinositol (PI) in the resurrection plant *Craterostigma plantagineum* [66]. Interestingly, we also find that in *K. crenulatum* the CDP-diacylglyerol-inositol 3-phosphatidyltransferase (EC 2.7.8.11) is up-regulated, which is responsible for the conversion of CDP-diacylglycerol into phosphatidyl D myo-inositol. Moreover, enzymes in the diacylglycerol pathway such as digalactosyldiacyl-glycerol synthase (K09480) or enzymes leading to triacylglycerol [e.g. diacylglycerol O-acyltransferase (K00635) and phospholipid: diacylglycerol acyltransferase (K00679)] are up-regulated.

### Antioxidants

Antioxidant protection has been studied extensively in lichens and photobionts of lichens [67]–[69]. It has been found that the green photobionts contain protective enzymes such as superoxide dismutase, catalase, peroxidases, glutathione reductase and ascorbate peroxidase, in combination with non-enzymatic substances such as glutathione, α-tocopherol and ascorbic acid (recently summarized [4]). Increased glutathione levels have been determined to be a consequence of supplemental UV irradiation in *Chlorococcum infusionum* and *Chlorogonium elongatum* [70]. In *Trebouxia excentrica*, the photobiont of the lichen *Cladonia vulcani,* the reduced form of glutathione (GSH) is progressively oxidized to glutathione disulfide (GSSG) during a desiccation period of 20 days [68].

Our transcriptome data showed that contigs with similarity to the first step of glutathion biosynthesis, glutathione-S-transferase and glutathione peroxidase are up-regulated. The latter are involved in the protection of the cells from oxidative damage by reducing lipid hydroperoxides to alcohols and free hydrogen peroxide to water. In contrast, contigs with similarity to glutathione-degrading enzymes are down-regulated. However, we currently cannot explain why the glutathione-disulfide reductase which is responsible for reduction of GSSG to GSH is also down-regulated. To corroborate these findings, we have tested if elevated levels of glutathione could be found in *K. crenulatum*. We found a marked elevation (to 177% of the original value) as demonstrated by immunoelectron microscopy with polyclonal antibodies against glutathione [45]. It would have been interesting to determine the compartments which show the largest increases in glutathione levels. Unfortunately, the subcellular distribution, which showed the highest concentrations in mitochondria in control cells, was virtually impossible to determine in desiccated cells of *K. crenulatum*. The reason for this is probably that the cytoplasm appears condensed in desiccated *K. crenulatum* cells [17], as well as in other desiccated green algae (for a summary see [3]).

In concordance with the above findings, we measured a significant increase in the total antiradical power of *K. crenulatum* (this study, DPPH-test). This test is commonly used to determine the total antioxidant activity (e.g. [71], [72]). For example, in *Saccharina latissima,* threefold higher level of antioxidants has been detected in generative (sorus) *versus* vegetative tissue [71]. In the case of the brown macroalga, this was attributed to a high phlorotannin content, which was considered responsible for protection of the reproductive tissue. In the present study we found evidence for a rapid adjustment of free-radical scavengers such as ascorbate and glutathione, induced by desiccation stress.

Additional potent antioxidants are pigments such as the secondary carotenoid astaxanthin, which has been found to be accumulated, e.g. in the green alga *Haematococcus pluvialis* [73]. However, based on our transcriptome data, *K. crenulatum* apparently cannot synthesize astaxanthin. Nevertheless we observed a strong up-regulation of enzymes for other carotenoids, which might also have antioxidant activity.

## Conclusion

The present study revealed that a transcriptome approach is feasible for investigating a strong stress as imposed by the desiccation of *K. crenulatum*. Our results indicate that cells react to the desiccation stress, in attempting to re-establish their energy metabolism (up-regulation of contigs related to respiration and photosynthesis). Furthermore the response is surprisingly similar to that found in embryophytes (LEA proteins, ERD proteins, ROS protection, possibly accumulation of RFO osmolytes) and might also be regulated by the abscisic acid signaling pathway, highlighting the close relationship of streptophyte algae to land plants and supporting an important role for streptophyte algae in the adaptation process that eventually led to colonization of the terrestrial habitat.

## Supporting Information

Figure S1 **A CO_2_ assimilation rate, filter temperature and effective quantum yield at different relative air humidity levels during a desiccation period of 2.5 h (RH 20%).** Measurements were continued after a 21-h recovery period in culture medium. B CO_2_ assimilation rate and effective quantum yield of photosystem II at the beginning of the desiccation period (n = 5, 5 min of initial desiccation period, blue bars), at the end of the desiccation period of 2.5 h (n = 5, purple bars) and after a recovery period of 21 h in culture medium (n = 5, green bars). Statistical analyses were carried out by one-way ANOVA with Tukey test (p≤0.001), C Effective quantum yield (Yield II) at the same time points as in B.
(PPTX)

Figure S2 **RNA integrity: A Gel electrophoretic separation of isolated total RNA from** ***Klebsormidium crenulatum*** **control cells (K1, K2, K3) and 2.5 h over silica gel-desiccated samples (T1, T2, T3). 18 S and 25 S RNA bands are marked.** The same percentage of the isolated total RNA was loaded for each sample. B Electropherograms of corresponding total RNA samples as shown in (A). The 18 S and 25 S bands were marked when applicable. RNA integrity (RIN) numbers were calculated with Agilent 2100 Expert software and ranged between RIN 5.7 and RIN 5.8 in the control samples and between RIN 3.8 and RIN 4.2 in the desiccated samples. RNA quality was considered appropriate when two distinct peaks were visible (K1 to K3); samples with elevated baselines (T1 to T3), where two peaks were still visible, were considered partially degraded, but were also used for the analysis. Although sample T3 had the highest RIN of the desiccated samples (RIN 4.2), no cDNA library could be constructed from this sample.
(PPTX)

Table S1 **Module reconstruction.** KO identifiers were retrieved from the KEGG orthology database for the complete, up-regulated and down-regulated dataset used to reconstruct KEGG modules. Column A, B and C show the results for the complete (all), the up-regulated and the down-regulated data set, respectively. Column D displays the KO identifier and column E the name of the pathway or enzyme.
(XLSX)

Table S2 **Read statistics for NG-6357 K1 lib21278,NG-6357 K2 lib21279,NG-6357 K3 lib21280, NG-6357 T2 lib21282, NG-6357 T3 lib21283.**
(DOCX)

Table S3 ***Klebsormidium crenulatum*** **contigs up-regulated upon strong desiccation.** The white region displays the result of the expression analysis using the DeSeq program. The green and blue region give the accession number of the most similar protein in streptophytes (green) and chlorophytes (blue). Kcr-ID, sequence identifier; baseMean, mean normalized counts, averaged over all samples from both conditions; baseMeanA, mean normalized counts from condition A; baseMeanB, mean normalized counts from condition B; foldChange, fold change from condition A to B; log2FoldChange, the logarithm (to basis 2) of the fold change; pval, p value for the statistical significance of this change; padj; p value adjusted for multiple testing with the Benjamini-Hochberg procedure (see the R function p.adjust), which controls false discovery rate (FDR). Ath-ID *Aradopsis thaliana*; Mtr-ID, *Medicago trunculata*; Aca-ID, *Aquilegia coerulea*; Stu-ID, *Solanum tuberosum*; Mdo-ID, *Malus domesticus*; Ptr-ID, *Populus trichocarpa*; Egr-ID, *Eucalyptos grandiflora*; Tca-ID, *Theobroma cacao*; Ppa-ID, *Physcomitrella patens*; Sit-ID *Selaginella italica*; Cre_ID, *Chlamydomonas reinhardtii*; Csu_ID, *Cocomycxa subellipsoidea*; Mpu_ID, *Micromonas pusilla*; Olu_ID; *Ostreococcus lucimarinus*.
(XLSX)

Table S4 ***Klebsormidium crenulatum*** **contigs down-regulated upon strong desiccation.** The white region displays the result of the expression analysis using the DeSeq program. The green and blue region give the accession number of the most similar protein in streptophytes (green) and chlorophytes (blue). Kcr-ID, sequence identifier; baseMean, mean normalized counts, averaged over all samples from both conditions; baseMeanA, mean normalized counts from condition A; baseMeanB, mean normalized counts from condition B; foldChange, fold change from condition A to B; log2FoldChange, the logarithm (to basis 2) of the fold change; pval, p value for the statistical significance of this change; padj; p value adjusted for multiple testing with the Benjamini-Hochberg procedure (see the R function p.adjust), which controls false discovery rate (FDR). Ath-ID *Aradopsis thaliana*; Mtr-ID, *Medicago trunculata*; Aca-ID, *Aquilegia coerulea*; Stu-ID, *Solanum tuberosum*; Mdo-ID, *Malus domesticus*; Ptr-ID, *Populus trichocarpa*; Egr-ID, *Eucalyptos grandiflora*; Tca-ID, *Theobroma cacao*; Ppa-ID, *Physcomitrella patens*; Sit-ID *Selaginella italica*; Cre_ID, *Chlamydomonas reinhardtii*; Csu_ID, *Cocomycxa subellipsoidea*; Mpu_ID, *Micromonas pusilla*; Olu_ID; *Ostreococcus lucimarinus*.
(XLSX)
