# Supplementary figures and images for: Transcriptomics of Desiccation Tolerance in the Streptophyte Green Alga Klebsormidium Reveal a Land Plant-Like Defense Reaction
Source: PLoS One. 2014 Oct 23;9(10):e110630. doi: 10.1371/journal.pone.0110630 (PMC4207709; doi:10.1371/journal.pone.0110630)

## Slide 1
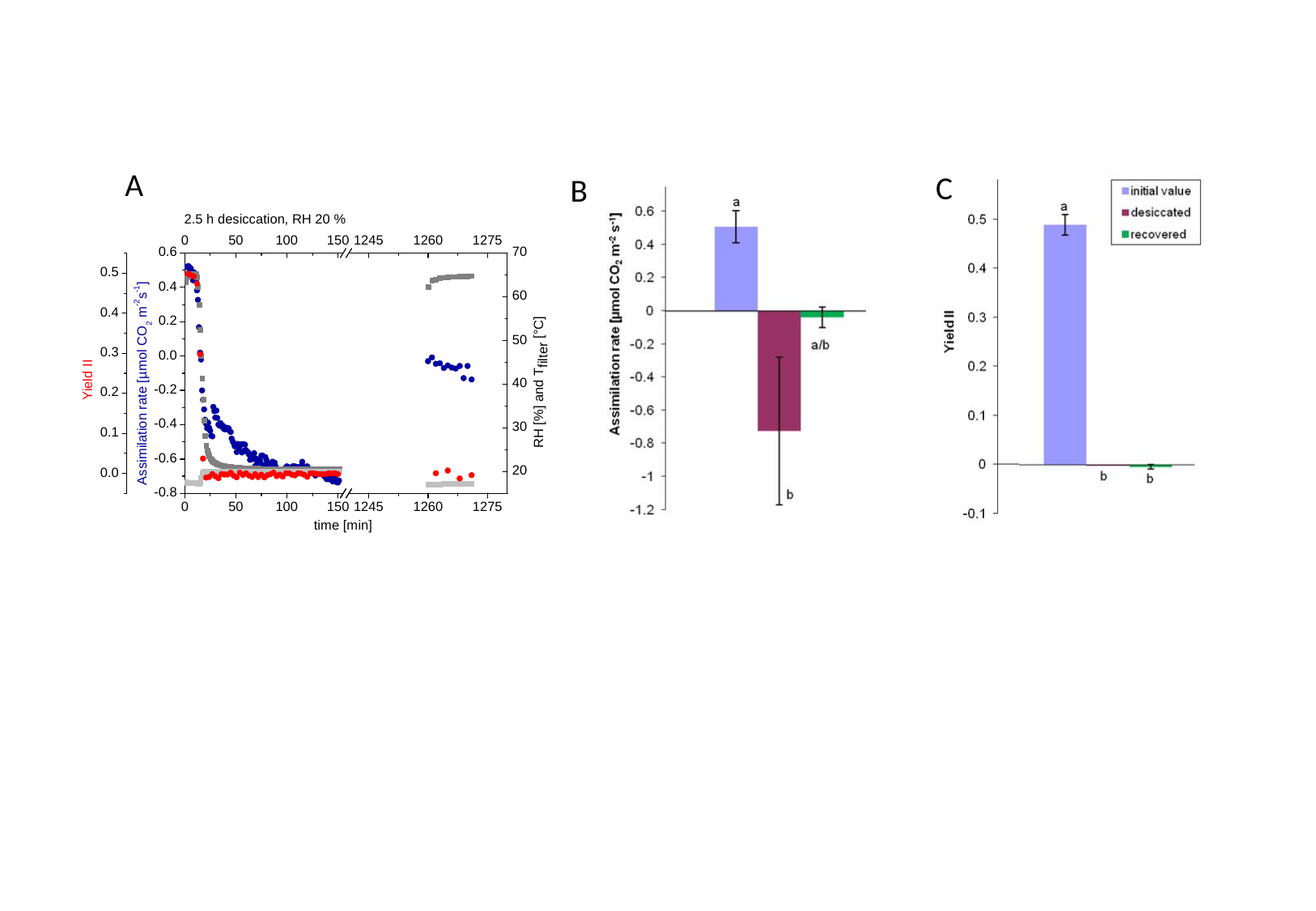

A
C
B

Supplement: Figure S1 — A CO2 assimilation rate, filter temperature and effective quantum yield at different relative air humidity levels during a desiccation period of 2.5 h (RH 20%). Measurements were continued after a 21-h recovery period in culture medium. B CO2 assimilation rate and effective quantum yield of photosystem II at the beginning of the desiccation period (n = 5, 5 min of initial desiccation period, blue bars), at the end of the desiccation period of 2.5 h (n = 5, purple bars) and after a recovery period of 21 h in culture medium (n = 5, green bars). Statistical analyses were carried out by one-way ANOVA with Tukey test (p≤0.001), C Effective quantum yield (Yield II) at the same time points as in B. (PPTX) [file pone.0110630.s001.pptx]

## Slide 1
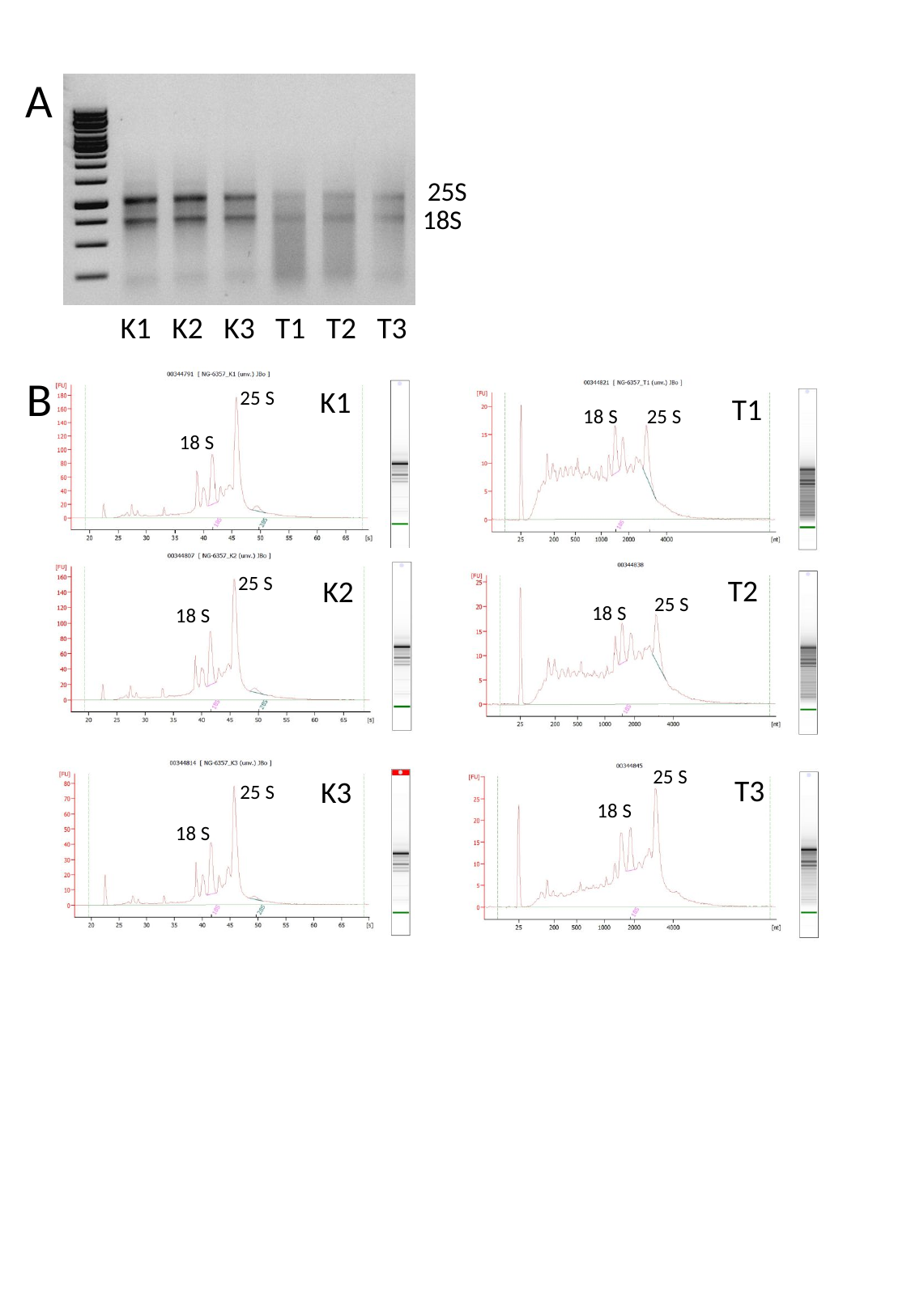

A
25S
18S
K1 K2 K3 T1 T2 T3
B
K1
25 S
T1
18 S
25 S
18 S
25 S
T2
K2
25 S
18 S
18 S
25 S
T3
K3
25 S
18 S
18 S

Supplement: Figure S2 — RNA integrity: A Gel electrophoretic separation of isolated total RNA from Klebsormidium crenulatum control cells (K1, K2, K3) and 2.5 h over silica gel-desiccated samples (T1, T2, T3). 18 S and 25 S RNA bands are marked. The same percentage of the isolated total RNA was loaded for each sample. B Electropherograms of corresponding total RNA samples as shown in (A). The 18 S and 25 S bands were marked when applicable. RNA integrity (RIN) numbers were calculated with Agilent 2100 Expert software and ranged between RIN 5.7 and RIN 5.8 in the control samples and between RIN 3.8 and RIN 4.2 in the desiccated samples. RNA quality was considered appropriate when two distinct peaks were visible (K1 to K3); samples with elevated baselines (T1 to T3), where two peaks were still visible, were considered partially degraded, but were also used for the analysis. Although sample T3 had the highest RIN of the desiccated samples (RIN 4.2), no cDNA library could be constructed from this sample. (PPTX) [file pone.0110630.s002.pptx]
