# Supplementary material for: Transcriptomics of Desiccation Tolerance in the Streptophyte Green Alga Klebsormidium Reveal a Land Plant-Like Defense Reaction
Source: PLoS One. 2014 Oct 23;9(10):e110630. doi: 10.1371/journal.pone.0110630 (PMC4207709; doi:10.1371/journal.pone.0110630)
Supplement: Table S2 — Read statistics for NG-6357 K1 lib21278,NG-6357 K2 lib21279,NG-6357 K3 lib21280, NG-6357 T2 lib21282, NG-6357 T3 lib21283. (DOCX) [file pone.0110630.s004.docx]

| Read category | NG-6357 K1  lib21278 |  | NG-6357 K2  lib21279 |  | NG-6357 K3  lib21280 |  | category NG-6357 T2  lib21282 |  | NG-6357 T3  lib21283 |  |
| --- | --- | --- | --- | --- | --- | --- | --- | --- | --- | --- |
|  | total | % | total | % | total | % | total | % | total | % |
| All | 20,993,787 |  | 30,885,085 |  | 24,507,245 |  | 26,363,212 |  | 12,292,923 |  |
| Mapped | 18,021,695 | 85.84 | 26,352,767 | 85.33 | 20,771,459 | 84.76 | 22,855,759 | 86.70 | 10,807,605 | 87.92 |
| Nonunique | 5,002,304 | 27.76 | 6,852,605 | 26.00 | 4.740.206 | 22.82 | 8,832,863 | 38.65 | 4,159,240 | 38.48 |
| Unique | 13,019,391 | 72.24 | 19,500,1621 | 74.00 | 16,031,253 | 77.18 | 14,022,896 | 61.35 | 6,648,365 | 61.52 |
| Duplicates | 7,049,989 | 54.15 | 11.072.429 | 56.78 | 8.569.523 | 53.46 | 11,155,960 | 79.56 | 3,981,374 | 59.89 |
| HQAligned | 5,969,402 | 33.12 | 8,427,733 | 31.98 | 7,461,730 | 735.92 | 2,866,936 | 12.54 | 2,666,991 | 24.68 |
